# Unilateral gustatory facial flushing in a child

**DOI:** 10.1016/j.jdcr.2023.07.004

**Published:** 2023-07-21

**Authors:** Mohammed Ameen, Fiona Lynch, Muriel Sadlier

**Affiliations:** Department of Dermatology, University Hospital Limerick, Co. Limerick, Ireland

A 2-year old boy presented with episodic right-sided facial flushing occurring immediately after eating. He had a history of an ipsilateral facial hematoma following forceps delivery which had healed without scarring. The flushing was first noted after the introduction of solid foods at 6 months of age. During mastication, right-sided linear erythema and warmth develops in the skin overlying the distribution of the auriculotemporal nerve (Fig 1), lasting approximately 1 hour. It occurs with all food-types and is not associated with sweating, pain, or pruritis. He is otherwise well with no developmental concerns.

**Fig 1:**
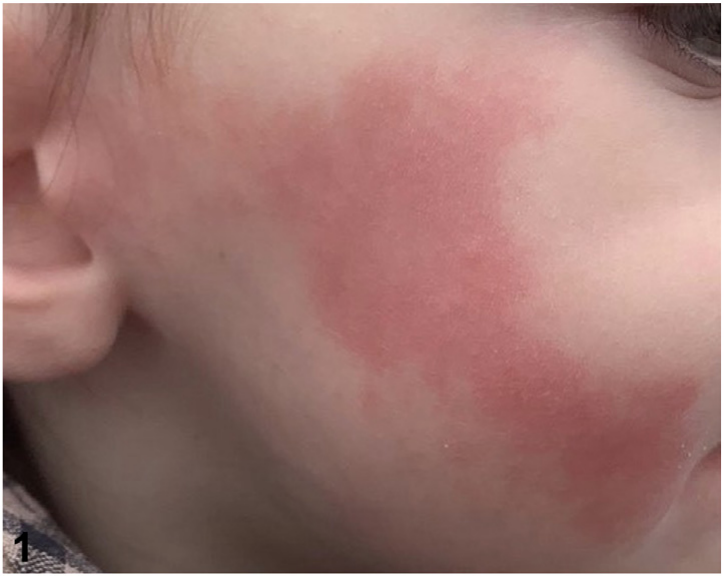

**Question 1: What is the most likely diagnosis?**

A. Harlequin syndrome
B. Mastocytosis
C. Frey syndrome
D. Food allergy
E. Trigeminal neuralgia

**Answers:**

A. Harlequin syndrome – Incorrect. Harlequin syndrome is hemi-facial flushing and hyperhidrosis which results from direct injury to the sympathetic fibers of the face. Symptoms are usually triggered by heat, exercise or strong emotions.^1^
B. Mastocytosis – Incorrect. Mastocytosis is a rare condition of uncontrolled mast cell proliferation, with the clinical presentation ranging from maculopapular skin rash to anaphylactic-like reactions.^1^
C. Frey syndrome – Correct. Frey syndrome (Auriculotemporal nerve syndrome) is a rare condition that usually results from direct injury to the auriculotemporal nerve, leading to gustatory flushing and/ or hyperhidrosis.^2^ It occurs due to aberrant regeneration of postganglionic parasympathetic neurons to the adjacent sweat glands and cutaneous blood vessels following injury.^3^ Frey syndrome is most commonly seen in adults, particularly following parotid surgery.^2^ In infants, Frey syndrome can occur following traumatic instrumental delivery, though familial cases have also been reported.^2^^,^^4^ Infantile Frey syndrome tends to present with gustatory flushing rather than hyperhidrosis.^2^
D. Food allergy – Incorrect. There are a few key features in this case that are inconsistent with the typical presentation of food allergy. Those include absence of a specific food trigger, as symptoms occur with all food types, absence of IgE-mediated systemic symptoms, and the spontaneous resolution of symptoms. Frey syndrome can often be falsely attributed to food allergy.^2^^,^^4^
E. Trigeminal neuralgia – Incorrect. While trigeminal neuralgia may cause unilateral flushing, it more commonly presents with episodic neuropathic pain that is triggered by physical contact.^1^ Absence of facial pain in this case makes the diagnosis less likely.

**Question 2: How to reach the diagnosis in this patient?**

A. The Minor starch-iodine test
B. Serum tryptase
C. Skin prick testing
D. Clinical history
E. Serum IgE level

**Answers:**

A. The Minor starch-iodine test – Incorrect. The Minor starch-iodine test is frequently utilized to detect gustatory hyperhidrosis. During the test, a combination of iodine and starch are applied over the affected facial area. Following that, a salivary stimulus is given to the patient. A blue-black discolouration over affected facial area becomes evident once excessive gustatory sweating occurs.^3^ As no gustatory sweating is reported in our case, the Minor starch-iodine test is not a suitable option.
B. Serum tryptase – Incorrect. Frey syndrome is not believed to be connected to mast cell activity and thus, measuring serum tryptase is not indicated.
C. Skin prick testing – Incorrect. This is not caused by food allergy.
D. Clinical history – Correct. Most cases of Frey syndrome are diagnosed based on appropriate clinical history and examination.^2^^,^^4^ The typical history of unilateral facial flushing post food ingestion, the absence of immunoglobulin E-mediated symptoms, the presence of underlying etiology such as traumatic instrumental delivery as in this case and the findings on examination make Frey Syndrome the most likely diagnosis.^2^^,^^4^ Minor starch-iodine test and infrared thermography can be used as confirmatory tests in certain cases.^2^^,^^3^
E. Serum IgE level – Incorrect. Serum IgE level can be useful to rule out true food allergy; however, it is not used to confirm the diagnosis of Frey syndrome.

**Question 3: What is the most appropriate treatment for this patient?**

A. Reassurance
B. Botulinum Toxin A injection
C. Diet restriction
D. Surgical transection of auriculotemporal nerve
E. Topical antiperspirants

**Answers:**

A. Reassurance – Correct. In uncomplicated infantile Frey syndrome not caused by facial surgery, reassurance is considered the mainstay treatment.^2^^,^^4^ This involves explaining the benign nature of the diagnosis and discouraging further tests. No specific treatment has been proven to be effective.^5^ Total resolution of symptoms or spontaneous regression tends to occur in nearly 75% of affected children.^4^
B. Botulinum toxin A injection – Incorrect. Intracutaneous injection of Botulinum toxin A has been widely used to help controlling gustatory sweating. This form of treatment has not proven to be effective, and the current level of evidence is confined to case reports and short case series.^3^
C. Diet restriction – Incorrect. As gustatory flushing and hyperhidrosis in Frey syndrome are not triggered by specific food type, diet restriction is not required.
D. Surgical transection of auriculotemporal nerve – Incorrect. This surgical approach is not commonly performed as it carries an increased risk of facial nerve injury. Other complications include poor cosmetic results and high likelihood of symptom recurrence. This approach is reserved for complicated cases that are refractory to conservative management.^3^
E. Topical antiperspirants – Incorrect. Topical antiperspirants can help with gustatory sweating; however, they are ineffective in fully eradicating the symptoms as their effect typically lasts for less than a day.^3^

## Conflicts of interest

None disclosed.
